# If I'll win it, I want it: The role of instrumental considerations in explaining public support for referendums

**DOI:** 10.1111/1475-6765.12358

**Published:** 2019-10-14

**Authors:** HANNAH WERNER

**Affiliations:** ^1^ Department of Political Science University of Leuven Belgium

**Keywords:** process preferences, referendums, participation, political decision making

## Abstract

Across established democracies, citizens express high levels of support for decision making via referendums. What drives these preferences remains yet unclear. In this article it is argued that, first, process preferences are less stable than previously assumed but vary substantially across policy proposals. Second, it is suggested that instrumental considerations play an important role in shaping citizens’ preferences for referendums. Specifically, citizens who favour the policy proposal or believe that they hold a majority opinion are expected to express more support for the use of referendums. An original survey was designed and conducted in the Netherlands (N = 1,289) that contains both between and within respondent variation across a range of policy proposals. The findings support these arguments: Both the desire for a specific policy change and the perception of being in the majority with one's policy preference relate to support for the use of referendums across policy proposals, levels of governance, and between and within respondents. This study contributes to a better understanding of process preferences by showing that these preferences have a non‐stable component and that instrumental considerations play an important role in citizens’ support for referendums.

## Introduction

Decision making via referendums is popular among citizens in established democracies, as has been repeatedly shown by public opinion surveys (Dalton et al. 2001; Bowler et al. 2007; Bengtsson & Mattila 2009; Neblo et al. 2010; Webb 2013; Font et al. 2015; Schuck & De Vreese 2015). For instance, in 2012, the European Social Survey (ESS) documents that 72.2 per cent of respondents across Europe give a score of eight or higher out of ten when asked how important it is for democracy that citizens have the final say on political issues by voting directly in referendums (ESS 2012). What drives support for referendums? Understanding the determinants of these preferences is crucial because broad public support for referendums is often interpreted in academia and political practice as a reason to adapt the political structures to cater to this apparent public demand for direct citizen involvement (e.g., Donovan & Karp 2006; Dalton & Welzel 2014; Dean 2016).

In the emerging literature on process preferences, support for direct decision making is often explained by either citizens’ higher normative expectations of democracy or – in contrast – by sheer frustration with the political elites (e.g., Bowler et al. 2007). Both explanations implicitly assume process preferences to be rather stable attitudes that reflect broader views on democracy or the state of the political system. The argument developed in this article is that process preferences do not merely consist of stable, normative ideas about how democracy should work. I argue that citizens’ support for decision making via referendums can vary substantially across policy proposals. These variations can be explained by instrumental considerations. I theorise that citizens think about whether a referendum will increase their chances of achieving the policies they desire. As a result, I expect citizens to be especially supportive of decision making via referendums on a given policy proposal if they either have a strong desire for the proposed policy change or if they believe they are in the majority with their opinion on this proposal.

Despite the fact that the importance of instrumental considerations has been widely acknowledged in political science, this factor has remained understudied in research on process preferences. I aim to shed light on the role of such considerations by building on research on instrumental reactions to electoral loss in the American context (Bowler & Donovan 2007; Smith et al. 2010) and on research on the role of outcome favourability for the evaluation of decision‐making processes (e.g., Esaiasson et al. 2016).

The argument is put to an empirical test by using an innovative survey design that can offer insights into the proposed non‐stable component of support for referendums. Respondents were questioned on their support for referendums, preferences and majority perceptions across a wide range of policy proposals at the national and local levels (data collected in the Netherlands, N = 1,289). The results of the within‐between random effects analysis allow to better understand the variations in support for referendums *between* individuals and the variations in support for referendums on different policy proposals *within* the same individual (e.g., Bell & Jones 2015). In particular, the within‐analysis provides a strong test of the causal argument, as alternative explanations for the support for referendums are held constant within the individual.

The results provide broad and consistent support for the instrumental hypothesis. Across levels of governance and policy proposals, preference for policy proposals and perceptions of being in the majority are associated with higher support for referendums between and within respondents. These findings open up exciting avenues for future research and contribute to a more detailed understanding of the determinants of process preferences by showing that these preferences vary across policy proposals and are influenced by outcome preferences and expectations.

## Explanations for support for direct decision making by citizens

How should political decisions be made from the viewpoint of contemporary citizens in established democracies? In recent years a body of scholarly literature has emerged studying individuals’ preferences for political decision‐making processes and documents a high amount of support for direct decision making such as through referendums. In the current debate, two prominent approaches to explain such broad support can be identified. The first notion of citizens’ process preferences is best reflected in the literature on political cultures which describes the emergence of ‘assertive citizens’ (Dalton & Welzel 2014), ‘self‐actualising citizens’ (Bennett 2008) or ‘critical citizens’ (Norris 1999) in established democracies. The argument goes that societies move towards a higher level of emancipative, self‐expressive and postmaterialist values. Citizens have become increasingly critical of political structures as their political resources grow as a result of rising education levels and greater access to information (Norris 2011). For instance, Dalton and Welzel (2014) have argued that this new generation of assertive citizens has higher egalitarian expectations of the political system and specifically calls for institutionalised involvement of the ordinary citizen. Thus, dissatisfaction with old structures and a more critical mind‐set evoke a desire for alternatives because ‘existing channels for participation fall short of democratic ideals’ (Norris 1999: 27; see also Hooghe et al. 2017). Different specifications of this approach have been suggested under labels such as ‘cognitive mobilisation’, ‘new politics’ or the ‘dissatisfaction hypothesis’ (Dalton et al. 2001). They all share the basic assumption that citizens have high democratic aspirations which spark genuine support for new opportunities for citizen involvement (Norris 2011).

The second approach opposes this notion. As Hibbing and Theiss‐Morse (2002) most famously argued, citizens are not at all in favour of more participation – they have simply become so deeply frustrated with current politics that they would choose almost any alternative. In the preference ranking of those citizens, decision making by benevolent politicians or experts is most desirable. If, however, politicians are primarily focused on their own interests and do not focus on the common good, citizens must have the opportunity to step in and exert control over them. More citizen involvement is therefore considered a second‐best option, a ‘medicine they must take in order to keep the disease of greedy politicians and special interests from getting further out of hand’ (Hibbing & Theiss‐Morse 2002: 131). Accordingly, support for direct democratic processes stems primarily from citizens’ general frustration with the establishment (Dalton et al. 2001; Bowler et al. 2007; Neblo et al. 2010; Schuck & De Vreese 2015).

Even though both accounts challenge each other in their substantial implications and their relation to normative accounts of participatory democracy, they do share some assumptions. First, they assume that support for direct democratic processes is relatively stable within individuals at a certain moment in time, depending on citizens’ values or general frustration with politics. As such, we would expect citizens to hold stable preferences for referendums across policy proposals. Second, in both approaches, the preference for referendums is assumed to emanate from general ideas about how democracy should work and focus solely on the process through which decisions ought to be made.

The argument put forward in this article challenges these assumptions. I argue that citizens’ process preferences are more dynamic than previously assumed. It is plausible that citizens have a baseline attitude towards referendums which is shaped by values and evaluation of politicians generally. However, I argue that process preferences also have a non‐stable, context‐dependent component that has so far remained understudied (see also Wojcieszak 2014; Dean 2016). Furthermore, to explain support for the use of referendums, I propose to look beyond general values and frustration and take instrumental considerations into account. Citizens care about the outcomes of political decision making and the expectation of such outcomes can shape preferences for certain decision‐making arrangements. Generally, context variation and instrumental considerations have remained largely neglected in the debate on process preferences. This is especially surprising as these considerations receive a substantial amount of attention in other research on political behaviour (e.g., rational choice approaches to voting). However, there are some notable exceptions that this article builds on that will be reviewed in the following section.

### Previous findings on the variability of process preferences and the role of instrumental considerations

Wojcieszak (2014) studied process preferences in Spain for three different issues by drawing upon the distinction made by Carmines and Stimson (1980) between easy and hard issues. She found that particularly for the ‘easy’ issue of abortion, support for decision making by citizens is highest, followed by the issue of migration and the ‘hard’ issue of taxation. Similarly, in his qualitative interviews (Q‐method) with citizens and civil servants, Dean (2016) found differences in support for citizen involvement depending on issue area.

Instrumental considerations are rarely discussed in the literature on process preferences, yet one notable exception is Smith et al. (2010). Inspired by Bowler and Donovan's (2007) study on the relationship between electoral loss and support for institutional reform, Smith et al. studied support for a national referendum in the American context. Using panel data, they showed that support for a national referendum increases directly after an election if participants lost in the election. The authors explain these findings by arguing that there are ‘strategic pockets of support (and opposition) for a national referendum’ (Smith et al. 2010: 509). Because citizens know that they have less chance of getting their desired outcome through representative decision making if their party is not in power, it is ‘rational’ to be more in favour of referendums to reach desired policies. Notwithstanding this study's important contribution, further evidence is required to comprehensively test the existence of instrumental considerations in process preference formation because there are various explanations for why losing in elections and support for alternative forms of decision making can be linked (such as retrospective denunciation of the electoral process; see Daniller 2016).

Instrumental considerations were also found to be of importance for related topics, such as support for national primaries after elections in the United States (Tolbert et al. 2009), electoral reform (Bowler & Donovan 2007; Aldrich et al. 2014; Blais et al. 2015) or support for institutional change among politicians (Bowler et al. 2006). Finally, another strand of literature has documented that evaluations of political procedures are influenced by their outcome favourability (e.g., Arvai & Froschauer 2010; Esaiasson et al. 2016; Arnesen 2017; Marien & Kern 2018) . This study aims to expand this notion by arguing that the same holds true for the anticipation of outcomes when thinking about preferred decision‐making procedures. To what extent this expectation holds empirically is unclear to date.

### A neglected determinant of process preferences: Instrumental considerations

There are two main arguments in this article. First, the assumption that citizens hold only stable, all‐encompassing preferences has to be qualified. I argue that citizens’ preferences for referendums also entail a non‐stable component which leads to support for decision making via referendums in some cases but not in others. Rather than being merely principled supporters or opponents of referendums, citizens might switch from referendum supporter to referendum opponent based on the policy proposal at hand. This notion of variability of process preferences does not compete with or disqualify existing explanations for support for direct democratic decision making. I do not argue that broader democratic aspirations or general dissatisfaction play no role. In fact, it seems most plausible that citizens’ process preferences consist of a stable and non‐stable component.

Second, I argue that individual expectations of achieving desired policy outcomes play a substantial role in shaping preferences for referendums. According to this instrumental explanation, citizens would prefer referendums to be used to make political decisions when they perceive that this process is likely to yield more favourable outcomes. I expect such instrumental considerations to manifest themselves in two ways. First, I expect citizens that support specific policy proposals that entail a shift away from the *status quo* to be more supportive of decision making via referendums on these proposals. The argument here is that citizens who desire a specific deviation from the *status quo* have nothing to lose by demanding a referendum on such a proposal but could potentially gain the desired change. In contrast, citizens who do not support the proposal but would rather stick to the *status quo* or favour a different kind of policy change have nothing to gain from such a referendum but potentially much to lose (for a similar argument with regard to voting in referendums, see Schuck & De Vreese 2009). Therefore, I expect particularly the proponents of a policy proposal to favour a referendum on this proposal.^1^ The second and related expectation is that citizens who believe they are in the majority with their opinion on a given proposal will be more supportive of decision making via a referendum. Here the assumption is that citizens evaluate how likely it is that they might win a referendum and adapt their support for this decision‐making arrangement accordingly.^2^

These two effects can be specified on two different levels. First, I expect that citizens who favour a specific policy proposal that moves away from the *status quo* are more supportive of making a decision on this proposal via a referendum than citizens who favour this specific policy proposal less. I expect that citizens who perceive they hold the majority position to be more supportive of making a decision on this proposal via a referendum than citizens who believe they are not in the majority. As such, I expect differences *between* citizens (i.e., between individuals effects).

*H1a*: Individuals who favour policy proposals that entail a shift away from the *status quo* are more supportive of the use of a referendum on these proposals than individuals who are less in favour of these policy proposals.
*H2a*: Individuals who believe they hold the majority on specific policy proposals are more supportive of the use of a referendum on these policy proposals than individuals who believe they do not hold a majority.

Second, I expect that citizens can prefer different decision‐making processes depending on the specific policy proposal at stake. Hence, in addition to the differences in process preferences *between* individuals, I expect that someone can hold a diverse set of process preferences depending on the policy proposal that is up for decision (i.e., differences *within* individuals). I argue that this non‐stable component of process preferences can be, at least in part, explained through instrumental considerations. I expect that someone is more supportive of decision making by referendum if she or he is in favour of the specific policy change to be decided or thinks his or her position on this proposal is the majority position. Accordingly, I also assume individuals hold different preferences across different policy proposals (within individual effects).

*H2a*: Individuals are more supportive of the use of a referendum on a policy proposal when they favour the proposal compared to when they favour the proposal less.
*H2b*: Individuals are more supportive of the use of referendum on a policy proposal when they think they are in the majority compared to when they think they are in the minority.

This approach differs from the current explanations in its assumption of stability of process preferences and its outcome orientation. It implies that rather than merely procedural characteristics, citizens also take into account the actual outcome of a referendum. The assumption is that citizens take political decision making seriously for what it eventually does: allocating scarce resources. While the term ‘instrumental’ may imply a focus on personal gain, this argument refers more broadly to policy preferences, whether they are egotropic or sociotropic.

## Design

To put the proposed argument to an empirical test, a design was required that contains data on for analysis both between and within individuals to investigate the existence of a non‐stable component in process preferences and explain it with the suggested instrumental considerations. To this end, survey data was collected to retrieve information on the preferences for referendums and policy proposals as well as majority perceptions across a range of policy proposals (ten) on the national and the local levels among 1,289 Dutch citizens. Collecting data on the same individual across various proposals enables me to study whether the same individual supports decision making via referendums on some proposals but not on others, and to what extent this variation can be explained by support for specific policy change and perceptions of being in the majority. The additional advantage of designing a within component is that confidence in the causal argument can be strengthened. Since all other individual‐level factors are held constant in such an analysis (such as demographic characteristics or political attitudes), all confounding variables at the individual level can be ruled out by design.

In the Dutch context, advisory initiative referendums on the national level were legally possible at the moment of data collection.^3^ Even though nationwide referendums occur rarely (so far only three; Müller 2018), advisory referendums at the local level are more frequent (193 referendums between 1906 and 2014; Van der Krieken 2015). This indicates that citizens are familiar with the institutional tool of a referendum and find its use credible. Accordingly, the Netherlands presents a rather representative case for most European countries where referendums occur from time to time but are not a frequent part of everyday political decision making as is the case, for instance, in Switzerland (Qvortrup 2014). As Jacobs (2018) points out, it is crucial for the study of referendums to move beyond the usual suspects that hold regular referendums such as Switzerland or the United States. The potential for generalising from this case to other countries will be addressed in the discussion section.

### Data

Data was collected with the help of an online‐panel company (PanelClix) in July 2017. PanelClix employs a stratified sampling approach to reach population distributions on age, gender, education and region.^4^ The obtained sample consists of 1,289 Dutch citizens with an average age of 51 years; 44 per cent are female, 35 per cent hold a college degree and the respondents come from 334 different Dutch municipalities. According to census data, the sample represents the Dutch population that is eligible to vote well (>18 years old), yet men are slightly over‐represented.^5^ To avoid over‐sampling of politically interested and engaged citizens, the invitation referred merely to ‘decision making’ as the topic of the survey. Respondents that raced through the questionnaire and exerted impossible response times (less than 25 per cent of the average response time^6^) were excluded from the analysis.^7^

### Measures

To measure preferences for the use of referendums, previous research has often simply asked respondents if they desire ‘more direct democracy’ or ‘more referendums’ without being particularly specific about the actual procedure this would entail. As a result, both quantitative and qualitative research suggest that citizens have limited and different understandings of such broad approaches to political decision‐making arrangements (e.g., Craig et al. 2001). There are several forms of referendums that can potentially be desirable to citizens to a different extent. Therefore, it was necessary to provide participants with a clear description of what was meant by ‘referendum’ in the context of this survey, even though this comes at the expense of only collecting data about one of various potential types of referendums (for a discussion of this trade‐off, see Dean 2016). Accordingly, before answering questions on their process preferences, participants read a short description of the type of referendum they were being questioned about:

In the Netherlands, political decisions are usually taken by elected representatives. But in many countries and also in the Netherlands, there is a debate about whether more decisions should be made by citizens directly, for instance via *binding referendums*. In a referendum all citizens can vote on a specific issue. Binding means that the government has to follow the result of the referendum. The outcome of a referendum is only valid if a critical mass of people take part, typically 30%.

Afterwards, respondents were asked if they are generally in favour of using such binding referendums at the national level when important political decisions are to be made. The answer options ranged from completely against (1) to completely in favour (7). This question was adapted to measure preferences for the local level and across specific policy proposals at the national and local levels. The proposals upon which respondents were questioned are presented in Table 1. The goal was to achieve a diverse set of proposals to prevent the findings being driven by a particular issue family. The choice of policy proposals at the national level was guided by what issues are considered (somewhat) relevant by the Dutch population based on survey data from the Eurobarometer (European Commission 2017) while also including specific proposals that were present in the public debate at the time of the survey, such as the debate on home care at the local level. Further, to build on previous research by Wojcieszak (2014), who found that citizens are more supportive of decision making by citizens on easy issues compared to hard issues (based on the distinction made by Carmines and Stimson (1980)), both issue areas were covered in this survey. Furthermore, proposals were selected that address different cleavages in society, such as high versus low income, low versus high medical needs, young versus old, culturally progressive versus culturally conservative to ensure variation on policy preferences.

**Table 1:** Policy proposals used in the study

National level	Local level
People with high incomes should pay more taxes We should drop the ‘own risk’ in health care payments More migrants should be allowed to come to the Netherlands Abortion should be allowed after the third month Unemployed people should receive more financial support Pension payments should be increased	Cars should be banned from the centre in [municipality] More police officers should be employed in [municipality] More houses in [municipality] should be reserved for social housing Home care should be publicly regulated in [municipality] instead of run by private companies

The main independent variables of interest are support for specific policy proposals that entail a shift away from the *status quo* and perception of being in the majority on this specific proposal. Support for the specific policy proposals was measured by asking the respondents whether they agreed with a number of proposals, which were all framed as deviation from the *status quo* (see Table 1). Answer options ranged from 1 (completely disagree) to 7 (completely agree). An overview of respondents’ support of these proposals can be found in Table A15 in the Online Appendix.

To assess *perceptions of being in the majority* for each of the presented policy proposals, respondents were asked whether they thought that most people in the Netherlands have the same opinion on the respective proposal as them, with answer options ‘yes’, ‘no’ and ‘don't know’. In addition, the general assessment of the overall belief of being in the majority was measured. To this end, individuals were asked to what extent they believed that citizens in the Netherlands/their municipality generally share their opinions. The scale ranged from 1 (not at all) to 7 (absolutely), including a don't know option.

The ordering of the questions was designed as follows: (1) preference for a referendum on the described policy proposal; (2) preference for these policy proposals; and (3) perceptions of holding a majority view on these proposals. This order was chosen to avoid guiding respondents in the direction of the theoretical argument. Further, respondents were first questioned on their general preferences and then on policy‐specific preferences. To avoid ordering effects, all items within the policy‐specific batteries were presented in random order.

### Control variables

The advantage of a within design is that it, by definition, controls for individual characteristics, such as political attitudes or demographics. This is because comparisons are made within the same individual and thus (possibly confounding) individual characteristics are kept constant.

For the between individuals part of the analysis, a range of variables that are typically found predictive in research on process preferences were included. First, as advanced by advocates of the cognitive mobilisation hypothesis, political interest and emancipative values are assumed to positively relate to the desire for more inclusive decision‐making procedures and were thus included. Political interest was measured with the standard ESS item (*How interested are you in politics?* 1–7, ESS 2017). A short battery assesses emancipative values comprising the PVQ^8^ measures of the Schwartz values of Universalism and Self‐direction (Beckers et al. 2012).

Second, drawing on the stealth democracy thesis, stealth democratic attitudes were measured using the scale proposed by Hibbing and Theiss‐Morse (2002). Further, political trust and other measures of political support have been shown to negatively relate to the preference for referendums (e.g., Bengtsson & Mattila 2009; Webb 2013; Dalton et al. 2001). Therefore, a standard measure of trust in institutions was included (*How much trust do you have in the following institutions? Parliament/politicians/political parties/legal system/police*, and for the local level: *City council in [municipality], local government in [municipality]?*, 1–7, ESS 2017). Risk aversion is included because it seems plausible that individuals who are more prone to taking risks are also less hesitant to risk direct democratic procedures (Bowler & Donovan 2007). Risk aversion was measured by asking: *Are you generally a person who is fully prepared to take risks or do you try to avoid taking risks?* (1–7; Dohmen et al. 2011). Level of education has been shown to negatively relate to preferences for direct democratic decision making (Anderson & Goodyear‐Grant 2010; Coffé & Michels 2014) and was therefore included. Finally, respondents were questioned on their age, gender and generalised trust (*Most people can be trusted*, 1–7, ESS 2017). The complete questionnaire as well as descriptive information on the measures can be found in Online Appendix A.

### Analysis strategy

To study the hypothesised relationships on the within and the between levels, the data is reshaped into a long format. This yields a clustered dataset with responses to the different proposals nested within respondents. I use regression models with clustered robust standard errors to analyse the data. Employing a multilevel regression approach yields substantially the same results (see Table C2 in the Online Appendix).

The study was designed to estimate effects *between* and *within* respondents. Accordingly, an analysis strategy was required that is able to distinguish between effects both between and within individuals. Such a method, referred to as the unified within‐between random effects (RE) framework, has been developed for analysing nested or panel data (Bell & Jones 2015; for a recent empirical application, see De Blok et al. 2017). To separate within effects from between effects, the variance of the independent variable is decomposed into a respondent‐specific mean value (between respondent effect) and a value for the difference between the actual score and the respondent‐specific mean value. This framework allows the estimation of within‐ and between‐level effects separately and combines the advantages of both fixed and random effects by modelling causal heterogeneity instead of simply controlling for it (for further information on the method, see Bell & Jones 2015; De Blok et al. 2017).

## Results

**Figure 1:**
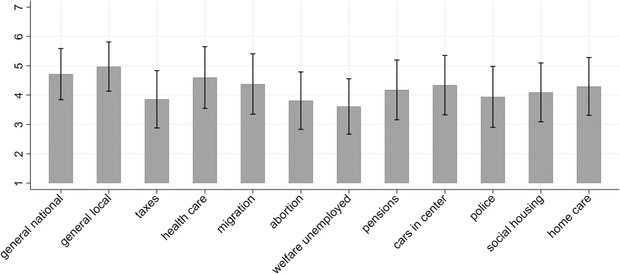
Support for the use of referendums across policy proposals and levels of governance. Notes: Mean scores, N = 1,289, error bars represent standard deviations.

Before turning to the core analysis of this article, descriptive information on the main dependent and independent variables of interest are presented. First, the overall support for the use of binding referendums is quite strong (see Figure 1). On the national level, the mean on a seven‐point scale is 4.7 with 60 per cent of people being rather or completely in favour of binding referendums. On the local level, support is even slightly stronger, with a mean of 4.9 and 65 per cent of participants being rather or completely in favour of binding referendums. This is in line with findings from large‐n surveys such as the ESS (2017) or the LISS panel^9^ for the Dutch context (e.g., in 2012, 62.55 per cent supported the use of referendums). Overall, support for referendums on specific policy proposals is lower with substantial variation across proposals and between individuals (as the standard deviations indicate).

Second, the stability of process preferences across policy proposals is examined. To this end, respondents are categorised into three groups: stable supporters, stable opponents and switchers (with a conservative estimate of switchers). As can be seen in Table 2, the predominant majority of respondents fall into the switcher category, strongly indicating that preferences for referendums contain a non‐stable component.^10^ Hence, there seems to be more to citizens’ preferences for the use of referendums than a stable attitude about how political decisions should generally be made.

**Table 2:** Types of referendum supporters

	Stable opponents	Switchers	Stable supporters
N	200	832	257

Notes: Full opponents are individuals that are neutral or against referendums across all policy proposals; full supporters are individuals that are neutral or in favour of referendums on all policy proposals; and switchers are all the others. N = 1,289.

### The relationship between preference for proposed policy change, majority perceptions and support for the use of referendums

**Figure 2:**
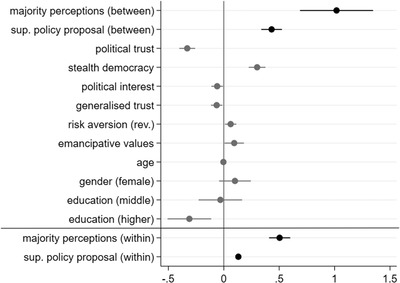
Explaining support for the use of referendums between and within respondents. Notes: R^2^ = 0.25; N = 8847. Non‐standardised coefficients are presented; confidence interval (CI) = 95 per cent; ‘don't knows’ are excluded from the analysis; the estimates are the results of an ordinary least squares regression with clustered robust standard errors (proposals nested within individuals). The corresponding table is Table B1 in the Online Appendix.

To test the main argument of this study, an OLS model with clustered robust standard errors is run, including the previously described between and within components for the two independent variables of interest. Figure 2 plots the unstandardised regression coefficients of this analysis both for the variables at the within and between levels (all control variables are at the between level). In Figure 2, we see at the between level that both the preference for the proposed policy change and perceptions of being in the majority have positive and significant effects on support for decision making via referendums across all ten different policy proposals. This means that individuals who support the proposed policy change or believe their opinion is in the majority exert higher levels of support for decision making by referendums on these proposals than individuals who have a lower desire for the proposed policy change or perceive themselves to be in the minority. The same substantial results are obtained by running bivariate analyses for the individual independent variables without controls (see Table C4 in the Online Appendix). Hence, *H1a* and *H2a* are supported by the data. These findings on the between level support the general argument of instrumental considerations, but despite controlling for other predictive factors, alternative explanations cannot be ruled out completely at this point.

Turning to the within level, the model controls for all between respondent differences and therefore can confidently rule out alternative explanations. As can be seen in Figure 2 we also see that the preference for policy change and majority perceptions have positive and significant effects at the within level. Accordingly, the more in favour one is of a specific policy proposal that entails a shift away from the *status quo*, the more supportive one is of decision making via a referendum on this proposal. If one thinks one holds a majority position on a specific policy proposal, one is more supportive of decision making via a referendum on this policy proposal.

The same results are obtained running a fixed effects model for the within level only (see Table C5 in the Online Appendix). These findings support *H1b* and *H2b*. Excluding these instrumental explanations from the model results in a drop in R^2^ from 0.25 to 0.17, indicating a substantial explanatory power of the independent variables of interest.

### Scope conditions

To gain more insights into the scope conditions of the effects at play, additional analyses are run including moderators. Given that these analyses are post‐hoc tests, these results and particularly the obtained significance values must be interpreted with caution. First, majority perceptions could be more influential when the preference for a specific policy proposal is strong and vice versa. However, these possible interaction effects at both the between and within levels are not significant (see Table C6 in the Online Appendix). Thus it seems that majority perceptions and support for specific policy proposals operate independently of each other.

Second, the magnitude of the majority perceptions effect on process preferences might depend on the public salience of an issue. Based on Eurobarometer data (European Commission 2017) most proposals studied can be classified as related to debates that are either highly, moderately or barely important to the Dutch public at the moment of surveying. Including this salience variable as a moderator of majority perceptions results in positive interaction effects both at the between and within levels (see Table C7 in the Online Appendix). This provides first indications that majority perceptions are particularly important as drivers of support for decision making via referendums when the issue underlying a proposed policy change is salient in the public debate.

Third, the certainty individuals attach to their perceptions of being in the majority could also moderate the relationship at hand. To this end a measure of overall certainty is interacted with majority perceptions at the between level for national and local policy proposals. It does not seem that the effect is stronger or weaker for individuals that are less certain of their majority perceptions (see Tables C8 and C9 in the Online Appendix). However, while this first indication suggests that certainty is less important, the measure is rather coarse and cannot tap into within respondent variation.

Fourth, the level of education might moderate the relationships of interest at the within level. It is possible that highly educated people have more principled views on decision making via referendums and instrumental considerations play less of a role for them (see also Collingwood 2012). Indeed, the interaction analysis reveals that majority perceptions and support for specific policy change are more strongly associated with the preference for referendums on these proposals for lower educated respondents than for higher educated respondents (see Table C10 in the Online Appendix).

Fifth, both on the local and national levels there are substantial positive significant associations of perceptions of *generally* being in the majority and *general* support for the use of referendums (Table C11 in the Online Appendix).

### Robustness checks

Finally, the results of a range of tests are reported that assess the robustness of the main findings. First, I address the previously described idea that citizens’ process preferences consist of both a stable baseline component and a more dynamic, context dependent component. To this end, I include the general preference for referendums as a control variable in the model. As expected, the general preference does significantly predict support for referendums on specific policy proposals (see Table C12 in the Online Appendix). However, both at the within and the between levels we still see significant positive effects of policy‐specific instrumental considerations that barely change in magnitude compared to the main analysis (Figure 2). This finding lends support to the notion that specific process preferences are shaped both by a general preference for this decision‐making arrangement and by policy‐specific instrumental considerations.

Second, respondents with ‘don't know’ responses to the majority perceptions questions are taken into account. This cannot be done in the main within‐between random effects model due to the calculation of within and between variance, but can be included in a general OLS with clustered robust standard errors. The results show that respondents who stated that they did not know about the opinions of their fellow citizens do not differ from respondents who perceived they are in the minority, while the effect of perceptions of being in the majority remains positive and significant (Table C13 in the Online Appendix). This finding fosters confidence that it is indeed the perception of being in the majority that is positively related to support for referendums.

Third, to prevent the detected effects from being driven by one or two specific policy proposals, the main analysis is also run for each proposal separately (see Tables C14 and C15 in the Online Appendix). The majority perception effect can be found across all proposals but less so for the proposals related to taxation (also, p = 0.051) and social housing (not significant). The effect of favourable preferences on policy proposals is also robust across most proposals except cars in city centres (not significant) and immigration (significant negative effect). The latter can be explained by a strong correlation between being in favour of referendums generally and opposing more migration (see Table C16 in the Online Appendix). In short, it seems that the effects documented in the main analysis hold across most proposals and are not merely driven by a single proposal. The robustness checks showed that the documented effects hold across a range of additional specifications and analysis types.

## Discussion

Understanding the origins of citizens’ process preferences is crucial. Almost any discussion of the virtues of democratic innovations and increased citizen involvement is to some extent based on an apparent public demand for such decision‐making arrangements. Studying process preferences is thus not only relevant to scholars working in this field, but also because of the political influence of the observed desire for more direct democratic policy making across Europe (Smith 2009).

In this article, the argument was put forward that individuals take their outcome expectations into account when expressing support for political decision‐making procedures. To my knowledge, this study is the first to explicitly make this argument and provide a comprehensive test across different policy proposals, levels of governance, and between and within individuals. The results of the study show that preferences to use a referendum indeed vary across policy proposals. These preferences are significantly associated with support for a specific policy proposal and the perception of having the majority on one's side – which holds across proposals, levels of governance, and between and within respondents.

These findings have important implications for our understanding of citizens’ attitudes towards direct democratic decision making. Citizens seem to think more pragmatically about decision‐making procedures than previously accounted for in the literature. This means that process preferences are not (only) stable political attitudes, but also contain non‐stable, context‐specific components. In this study, the vast majority of respondents (65 per cent) was neither fully committed nor fully opposed to decision making by referendum but supported its use occasionally. It thus seems that categorising citizens as strictly ‘assertive citizens’ or ‘stealth democrats’ can only apply for a small minority of the citizenry. These findings echo a recent discussion by Dean (2016), who calls for ‘understanding different forms of participation not as alternative models of governance but as responses to specific problems’ (see also Warren 2017). The debate about process preferences might benefit from shifting the focus away from existing accounts of normative models of democratic decision making to a more context‐ and problem‐specific perspective. In this study, the existence of a non‐stable component in process preferences could be shown already by simply varying the policy proposals while keeping the broader political context constant. There are a multitude of other factors that can vary given a specific referendum and that can potentially impact citizens’ preferences, such as the initiator of the referendum, parties in power or question wording, to name but a few.

Second, the findings have important implications for the potential of referendums to foster democratic legitimacy. If the anticipation of policy outcomes is a core driver of preferences for citizen involvement, a crucial factor in determining satisfaction with the process and its outcomes is the accuracy of such anticipation. If majority perceptions are accurate, it might actually be good from a normative perspective if citizens that overwhelmingly desire a certain policy demand a direct tool to fulfil this preference. As such, referendums can be used as a shortcut to realising broadly supported policies without lengthy negotiations between parties. Presumably, scenarios with a strong majority across party lines do not occur too often. Examples could be sociocultural issues such as gay marriage or the right to abort pregnancy. From a perspective of increased legitimacy perceptions, individuals that correctly assumed they are in the majority, demand a referendum and subsequently win this referendum, will be satisfied as well (Marien & Kern 2018).

Yet, if outcome expectations do not align with the actual distribution of preferences in society, referendums could potentially even cause a decline in legitimacy beliefs. If the main reason to demand a referendum is a high expected utility, satisfaction will only be the consequence if the outcome lives up to the expectations. The implementation of a referendum could result in surprised losers that expected to win and eventually lead to frustration, especially among those that called for a referendum in the first place.

This could be of particular importance as it seems that especially those citizens that hold extreme views and support policy change will develop a more favourable attitude towards decision making via referendums and potentially advocate for it in public. This parallels the observation that referendums have recently received substantial support from populist parties on the fringes of the political spectrum (e.g., Mudde 2007; Bowler et al. 2017) and their supporters in Europe (Bjånesøy & Ivarsflaten 2016). Research on the motivation of politicians to introduce referendums have similarly found instrumental considerations such as maintaining or expanding power play an important role (Bowler et al. 2006; Ruth‐Lovell & Welp 2019).

As with every empirical investigation, this study does not come without limitations. Most importantly, it was cross‐sectional in nature, therefore limiting strong claims about causality. Yet, with regards to potential confounding variables, the within analysis provided strong evidence that it is indeed the perceptions of being in the majority and support for the specific policy proposals that elicit the effects, as all other variables were held constant within the individual. Whereas the within analysis is powerful in ruling out confounding variables, it cannot establish the temporal order criterion to establish the direction of causality. The only possible response to this objection in the context of this study is that it seems theoretically less plausible that individuals’ process preferences cause their perceptions of being in the majority or their policy preferences. Nonetheless, the present design cannot provide conclusive answers to these questions and experimental research on the topic is required to strengthen confidence in the causal argument. In addition, despite all the practical advantages of using the services of Online Panel companies, the stratified sampling approach (which does not include the whole population as a sampling frame) constrains our ability to make inferences about the general population. While this is particularly problematic when it comes to absolute scores and less so when focusing on the existence of a relationship between variables (Yeager et al. 2011), it would certainly be beneficial to replicate the study on a cross‐national probability sample.

Also the design comes with some limitations due to the balance between realism and maintaining a high level of control and parsimony of research design. While particular efforts were made to cover a wide range of issues, the argument regarding support for specific policy proposals and perceptions of being in the majority could play out differently for other issues that were not included in the present study – for instance, policy proposals in the exact opposite direction of the ones used in this study (e.g., decreasing taxes for higher incomes). This also affects the measurement of majority perceptions which could not grasp nuances between large or tight majorities or citizens’ certainty about their judgements. Furthermore, since the chosen policy proposals were hypothetical in nature, mapping on to broader societal questions, they were not as specific as real‐life referendum proposals. Particularly, the role that parties and partisanship play in the initiation of and public debate on a referendum (see, e.g., Jacobs et al. 2018) could not be taken into account in the present study.

Future studies could take up these factors and study them in conjunction. One example of a suitable design approach can be found in Arnesen et al. (2019), where they use conjoint experiments to uncover the conditions under which citizens consider referendums on EU membership legitimate.

What also demands discussion is the broader scope conditions of the present study. The theoretical argument was tested in the Dutch context where nationwide referendums occur rarely, like in most other European countries. Thus, referendums are still considered an unusual way of decision making that is only implemented if political will or popular demand is strong. Given the comparability of the Netherlands to other European countries in regards to frequency of referendums, I also assume the general mechanism to occur in most other European countries as well (indications are found in Dean (2016) and Wojcieszak (2014)). Yet future investigations in other national contexts are required. It is likely that the situation is different in countries such as Switzerland, where referendums are institutionally embedded in regular political decision making. It is plausible that a learning effect occurs when the alternation between winning and losing is experienced in the referendum process – particularly for citizens that hold inaccurate beliefs about being in the majority across policy proposals. Further, the degree to which instrumental considerations shape process preferences can change depending on other context factors. Particularly salient topics that are high on the public agenda could trigger instrumental considerations to play out, whereas this might be less so with less salient topics.

Despite these limitations, the present study provides a novel contribution to research on process preferences by shedding light on an understudied driver of support for referendums. Instrumental considerations shape citizens’ preferences for direct democratic decision‐making arrangements and these are more variable and context‐dependent than previously assumed.

## Notes

1 This is not to say that citizens could not also be supportive of other reforms or changes that would bring about the desired change, such as support for new elections or decision making by experts. As research has documented, citizens can be supportive of different (sometimes seemingly contradicting) processes simultaneously (e.g., Webb 2013).

2 Majority perceptions do not necessarily have to be accurate. I theorise the *perceptions* of being in the majority to matter and therefore make no assumption regarding citizens’ levels of sophistication and information.

3 In the following year, the law on constitutional referendums was abolished.

4 Quotas were soft and not interlocked.

5 The study was combined with another unrelated study on mobile app use of similar length. The average response time excluding the 1st and 99th percentile was 27 minutes.

6
*Statistics Netherlands CBS* (www.cbs.nl/nl‐nl/cijfers).

7 An additional analysis was conducted including these ‘racers’, yielding similar results. Results are presented in Table C1 in the Online Appendix.

8 Portrait Value Questionnaire, see Online Appendix A for wording.

9
*LISS Panel* (www.website.lisspanel.nl/).

10 For a table with further descriptive information about these groups, see Table C3 in the Online Appendix.
