# Supplementary material for: If I'll win it, I want it: The role of instrumental considerations in explaining public support for referendums
Source: Eur J Polit Res. 2019 Oct 14;59(2):312–30. doi: 10.1111/1475-6765.12358 (PMC7217211; doi:10.1111/1475-6765.12358)
Supplement: Supplementary file 1 [file EJPR-59-312-s001.zip › ejpr12358-sup-0001-Appendices.docx]

**APPENDICES**

“If I’ll Win It, I Want It. The Role of Instrumental Considerations in Explaining Public Support for Decision-Making through Referendums”

Contact: Hannah.werner@kuleuven.be

**APPENDIX A**

1. **Scales**

Note: The questionnaire was administered in Dutch. The original questionnaire is available upon request from the author

Note: [municipality] means that the name of the participants municipality was shown here (using qualtrics piped text)

- 1. **Support for the use of referendums**

Introduction text:

“In the Netherlands, political decisions are usually taken by elected representatives. But in many countries and also in the Netherlands, there is a debate about whether more decisions should be made by citizens directly, for instance via binding referendums. In a referendum all citizens can vote on a specific issue. Binding means that the government has to follow the result of the referendum. The outcome of a referendum is only valid if a critical mass of people take part, typically 30 %.”

Table A1. *General* s*upport for referendums*

| Item | Wording | Answer options | Mean | SD | Min | Max |
| --- | --- | --- | --- | --- | --- | --- |
| General support for referendums national | On the **national level,** are you generally in favor or against taking important decisions with **binding referendums** | - 1 Definitely against - 2 - 3 - 4 - 5 - 6 - 7 Definitely in favor | 4.71 | 1.74 | 1 | 7 |
| General support for referendums local | On the **local level,** are you generally in favor or against taking important decisions with **binding referendums** in [municipality] |  | 4.97 | 1.68 | 1 | 7 |

*Note:* N= 1289

Introduction text:

Below you can see a list of often-discussed issues on the national level. Do you think it should **be elected representatives** or citizens by means of a **binding referendum** that make the decision if…

Table A2. Proposal-specific support for referendums

| Item | Wording | Answer options | Mean | SD | Min | Max |
| --- | --- | --- | --- | --- | --- | --- |
| Taxes | people with high incomes should pay more taxes | - 1 definitely representatives - 2 - 3 - 4 - 5 - 6 - 7 definitely citizens in a binding referendum | 3.85 | 1.95 | 1 | 7 |
| Health care | we should drop the “own risk” in health care payments |  | 4.59 | 2.10 | 1 | 7 |
| Migration | more migrants should be allowed to come to the Netherlands |  | 4.37 | 2.06 | 1 | 7 |
| Abortion | abortion should be allowed after the third month |  | 3.80 | 1.95 | 1 | 7 |
| Welfare homeless | unemployed people should receive more financial support |  | 3.61 | 1.89 | 1 | 7 |
| Pension | pension payments should be increased |  | 4.17 | 1.03 | 1 | 7 |

*Note:* N= 1289

Below you can see a list of often-discussed issues on the local level. Do you think it should **be elected representatives** or citizens by means of a **binding referendum** that make the decision if…

Table A3. Proposal-specific support for referendums on the local level

| Item | Wording | Answer options | Mean | SD | Min | Max |
| --- | --- | --- | --- | --- | --- | --- |
| Cars in center | cars should be banned from the center in [municipality] | - 1 definitely representatives - 2 - 3 - 4 - 5 - 6 - 7 definitely citizens in a binding referendum | 4.33 | 2.02 | 1 | 7 |
| More police | more police officers should be employed in [municipality] |  | 3.94 | 2.07 | 1 | 7 |
| Social housing | more houses in [municipality] should be reserved for social housing |  | 4.09 | 2.01 | 1 | 7 |
| Home care | home care should be publicly regulated in [municipality] instead of by private companies |  | 4.29 | 1.96 | 1 | 7 |

*Note:* N= 1289

- 1. **preference for specific policy changes**

Introduction text

What is your personal opinion on these issues? To what extent do your agree or disagree with the following statements?

Table A6. Opinion on proposals

| Item | Wording | Answer options | Mean | SD | Min | Max | N |
| --- | --- | --- | --- | --- | --- | --- | --- |
| Taxes | people with high incomes should pay more taxes | - 1 completely disagree - 2 - 3 - 4 - 5 - 6 - 7 completely agree - Don’t know | 5.35 | 1.67 | 1 | 7 | 1269 |
| Health care | we should drop the “own risk” in health care payments |  | 5.52 | 1.79 | 1 | 7 | 1262 |
| Migration | more migrants should be allowed to come to the Netherlands |  | 2.98 | 1.77 | 1 | 7 | 1264 |
| Abortion | abortion should be allowed after the third month |  | 3.67 | 1.93 | 1 | 7 | 1199 |
| Welfare homeless | unemployed people should receive more financial support |  | 4.13 | 1.73 | 1 | 7 | 1251 |
| Pension | pension payments should be increased |  | 5.24 | 1.62 | 1 | 7 | 1236 |
| Cars in center | cars should be banned from the center in [municipality] |  | 3.80 | 2.15 | 1 | 7 | 1263 |
| More police | more police officers should be employed in [municipality] |  | 5.12 | 1.53 | 1 | 7 | 1222 |
| Social housing | more houses in [municipality] should be reserved for social housing |  | 4.95 | 1.69 | 1 | 7 | 1219 |
| Home care | home care should be publicly regulated in [municipality] instead of by private companies |  | 5.43 | 1.40 | 1 | 7 | 1197 |

Note: N=1289, don’t knows are excluded from descriptive statistics

- 1. **Majority perceptions**

Introduction text

If you think of other citizens in the Netherlands/ in [municipality], to what extent do you agree with the following statement?

Table A4. General majority perceptions

| Item | Wording | Answer options | Mean | SD | Min | Max |
| --- | --- | --- | --- | --- | --- | --- |
| Majority perceptions national | Most people in the Netherlands share my opinions | - 1 Completely disagree - 2 - 3 - 4 - 5 - 6 - 7 Completely agree - Don’t know | 4.76 | 1.32 | 1 | 7 |
| Majority perceptions local | Most people in [municipality] share my opinions |  | 4.87 | 1.33 | 1 | 7 |

*Note:* N= 1289

Introduction text

Do you think most people in the Netherlands share your opinion about the question if…

Table A5. Proposal specific majority perceptions on the national level

| Item | Wording | Answer options | Percent  Minority | Percent  Majority | Percent  Don’t know |
| --- | --- | --- | --- | --- | --- |
| Taxes | …people with high incomes should pay more taxes | - most people in the Netherlands **do not** share my opinion - most people in the Netherlands share my opinion - don’t know | 21.02 | 60.90 | 18.08 |
| Health care | …we should drop the “own risk” in health care payments |  | 14.04 | 73.62 | 12.34 |
| Migration | …more migrants should be allowed to come to the Netherlands |  | 36.54 | 44.30 | 19.60 |
| Abortion | …abortion should be allowed after the third month |  | 26.22 | 30.26 | 43.52 |
| Welfare homeless | …unemployed people should receive more financial support |  | 26.45 | 41.58 | 31.96 |
| Pension | …pension payments should be increased |  | 15.05 | 67.42 | 17.53 |

*Note:* N= 1289

Do you think most people in [municipality] share your opinion about the question if…

Table A5. Proposal specific majority perceptions on the local level

| Item | Wording | Answer options | Percent  Minority | Percent  Majority | Percent  Don’t know |
| --- | --- | --- | --- | --- | --- |
| Cars in center | …cars should be banned from the center in [municipality] | - most people in [municipality] **do not** share my opinion) - most people in [municipality] share my opinion - don’t know | 19.47 | 52.13 | 28.30 |
| More police | …more policemen should be employed in [municipality] |  | 9.08 | 63.93 | 27.00 |
| Social housing | …more houses in [municipality] should be reserved for social housing |  | 14.58 | 55.70 | 29.71 |
| Home care | …home care should be publicly regulated in [municipality] instead of by private companies |  | 9.70 | 60.82 | 29.48 |

*Note:* N= 1289

- 1. **Control variables**

Table A7. Trust in political institutions national

Introduction text: How much trust do you personally have in each of the following institutions?

| Variable | Wording | Mean | SD | Min | Max |
| --- | --- | --- | --- | --- | --- |
| Parliament | [country]’s parliament | 4.45 | 1.48 | 1 (no trust at all) | 7 (complete trust) |
| Parties | Political Parties | 4.72 | 1.40 | 1 (no trust at all) | 7 (complete trust) |
| Politicians | Politicians | 3.54 | 1.42 | 1 (no trust at all) | 7 (complete trust) |
| Legal system | Legal system | 3.63 | 1.53 | 1 (no trust at all) | 7 (complete trust) |
| Police | The police | 3.32 | 1.50 | 1 (no trust at all) | 7 (complete trust) |

*Note:* N= 1289

Table A8. Trust in political institutions local

Introduction text: How much trust do you personally have in each of the following institutions?

| Variable | Wording | Mean | SD | Min | Max |
| --- | --- | --- | --- | --- | --- |
| Municipal council | The municipality council in [municipality] | 4.27 | 1.33 | 1 (no trust at all) | 7 (complete trust) |
| Municipal government | The local government in [municipality] | 4.29 | 1.35 | 1 (no trust at all) | 7 (complete trust) |

*Note:* N= 1289

Table A9. Political interest

| Variable | Wording | Mean | SD | Min | Max |
| --- | --- | --- | --- | --- | --- |
| Political interest | To what extent are you generally interested in politics? | 4.55 | 1.50 | 1 (not interested at all) | 7 (very interested) |

*Note:* N= 1289

Table A10. Generalised trust

| Variable | Wording | Mean | SD | Min | Max |
| --- | --- | --- | --- | --- | --- |
| General trust | Do you think generally most people can be trusted or you can never be too careful? | 4.13 | 1.51 | 1 (you can never be too careful) | 7 (most people can be trusted) |

*Note:* N= 1289

Table A11: Emancipative values (using PVQ measure for Universalism and Self-direction)

Below we will describe some people. Please indicate to what extent you are similar or dissimilar to this person.

| Variable | Wording | Mean | SD | Min | Max |
| --- | --- | --- | --- | --- | --- |
| Universalism 1 | He thinks it is important that every person in the world be treated equally. He wants justice for everybody, even for people he doesn’t know. | 5.26 | 1.38 | 1(not at all like me) | 7(very similar to me) |
| Universalism 2 | It is important to him to listen to people who are different from him. Even when he disagrees with them, he still wants to understand them. | 5.43 | 1.39 | 1(not at all like me) | 7(very similar to me) |
| Universalism 3 | He strongly believes that people should care for nature. Looking after the environment is important to him. | 5.29 | 1.28 | 1(not at all like me) | 7(very similar to me) |
| Self direction 1 | Thinking up new ideas and being creative is important to him. He likes to do things in his own original way. | 4.67 | 1.44 | 1(not at all like me) | 7(very similar to me) |
| Self direction 2 | It is important to him to make his own decisions about what he does. He likes to be free to plan and to choose his activities for himself. | 5.30 | 1.28 | 1(not at all like me) | 7(very similar to me) |

*Note:* N= 1289

Table A12. Risk aversion

| Variable | Wording | Mean | SD | Min | Max |
| --- | --- | --- | --- | --- | --- |
| Risk aversion | How do you see yourself: are you generally a person who is fully prepared to take risks or do you try to avoid taking risks? | 3.94 | 1.39 | 1 (try to avoid risks) | 7 (fully prepared to take risks) |

*Note:* N= 1289

Table A13: Education

| Variable | Wording | Answer options | percent |
| --- | --- | --- | --- |
| Education | What is your highest level of education? | - Basisonderwijs (lower) | 1.33 |
|  |  | - Lager beroepsonderwijs (lower) | 5.73 |
|  |  | - Lager voortgezet onderwijs (VMBO, VBO, Mavo) (lower) | 12.39 |
|  |  | - Middelbaar beroepsonderwijs (MBO) (middle) | 30.35 |
|  |  | - Hoger voortgezet onderwijs (Havo, VWO) (middle) | 15.14 |
|  |  | - Hoger beroepsonderwijs (HBO) (higher) | 25.49 |
|  |  | - Wetenschappelijk onderwijs (WO) (higher) | 9.57 |

*Note:* N= 1289

Table A14: Stealth Democracy Index

| Variable | Wording | Mean | SD | Min | Max |
| --- | --- | --- | --- | --- | --- |
| SD_1 | Politicians would help the country more if they would stop talking and just take action on important problems. | 5.53 | 1.45 | 1(completely disagree) | 7(completely agree) |
| SD_2 | What people call ‘compromise’ in politics is really just selling out one’s principles | 3.94 | 1.71 | 1(completely disagree) | 7(completely agree) |
| SD_3 | Government would run better if decisions were left up to non-elected  independent experts. | 3.28 | 1.64 | 1(completely disagree) | 7(completely agree) |
| SD_4 | Government would run better if decisions were left up to successful  business people. | 4.43 | 1.62 | 1(completely disagree) | 7(completely agree) |

Table A15**:** *Distribution of preferences across policy proposals*

**APPENDIX B**

Corresponding tables to figures shown in paper

| Table B1. *Explaining support for the use of referendums between and within respondents* | | | |
| --- | --- | --- | --- |
|  | *coef* | *p* | *robust SE* |
| *within level:* |  |  |  |
| majority perception **within** (ref: no) | **.50** | **.000** | **.05** |
| sup. for policy proposal **within** | **.13** | **.000** | **.01** |
| *between level:* |  |  |  |
| majority perception **between** (ref: no) | **1.02** | **.000** | **.17** |
| sup. for policy proposal **between** | **.43** | **.000** | **.05** |
| Political trust | **-.33** | **.000** | **.04** |
| Stealth Democracy | **.30** | **.000** | **.04** |
| Political Interest | **-.06** | **.023** | **.03** |
| General trust | **-.06** | **.014** | **.03** |
| Risk aversion (reversed) | **.06** | **.018** | **.03** |
| Universal values | **.09** | **.035** | **.04** |
| Age | .00 | .157 | .00 |
| Gender (ref. male) | .10 | .170 | .07 |
| Education (ref. low) |  |  |  |
| middle | -.03 | .758 | .10 |
| high | **-.31** | **.002** | **.10** |
|  |  |  |  |
| Proposals (dummy, ref: home care) |  |  |  |
| cars in center | **-.68** | **.000** | **.07** |
| more police | **-.42** | **.000** | **.07** |
| social housing | **-.28** | **.000** | **.08** |
| taxes | **-.80** | **.000** | **.08** |
| own risk | -.12 | .147 | .08 |
| migrants | **.16** | **.042** | **.08** |
| abortion | **-.51** | **.000** | **.09** |
| welfare homeless | **-.83** | **.000** | **.08** |
| pension | **-.52** | **.000** | **.08** |
|  |  |  |  |
| R2 between | .40 |  |  |
| R2 within | .08 |  |  |
| R2 total | .25 |  |  |
| N | 8847 |  |  |
| *Note:* non-standardized coefficients are presented, estimates with a p-value below .05 are printed in bold. "don't know"s are exlcuded from analysis, estimates are the results of an ols regression with clustered robust standard errors (observations nested within individuals). | | | |

**APPENDIX C**

Additional Analyses

| Table C1: *Explaining support for the use of referendums between and within respondents including racers* | | | | | | |
| --- | --- | --- | --- | --- | --- | --- |
|  | | *coef* | | *p* | | *robust SE* |
| *within level:* | |  | |  | |  |
| majority perception **within** (ref: no) | | **.48** | | **.000** | | **.04** |
| sup. for policy proposal **within** | | **.13** | | **.000** | | **.01** |
|  | |  | |  | |  |
| *between level:* | |  | |  | |  |
| majority perception **between** (ref: no) | | **1.07** | | **.000** | | **.15** |
| sup. for policy proposal **between** | | **.45** | | **.000** | | **.04** |
| Political trust | | **-.29** | | **.000** | | **.03** |
| Stealth Democracy | | **.35** | | **.000** | | **.03** |
| Political Interest | | **-.08** | | **.001** | | **.02** |
| General trust | | **-.07** | | **.003** | | **.02** |
| Risk aversion (reversed) | | **.06** | | **.010** | | **.02** |
| Universal values | | **.09** | | **.014** | | **.04** |
|  | |  | |  | |  |
| Proposals (dummy, ref: home care) | |  | |  | |  |
| cars in center | | **-.58** | | **.000** | | **.06** |
| more police | | **-.35** | | **.000** | | **.06** |
| social housing | | **-.23** | | **.001** | | **.07** |
| taxes | | **-.72** | | **.000** | | **.07** |
| own risk | | -.08 | | .243 | | .07 |
| migrants | | .18 | | .012 | | .07 |
| abortion | | **-.46** | | **.000** | | **.08** |
| welfare homeless | | **-.74** | | **.000** | | **.07** |
| pension | | **-.46** | | **.000** | | **.07** |
|  | |  | |  | |  |
| R2 between | | .40 | |  | |  |
| R2 within | | .08 | |  | |  |
| R2 total | | .25 | |  | |  |
| N | | 10445 | |  | |  |
| *Note:* non-standardized coefficients are presented, estimates with a p-value below .05 are printed in bold. "don't know"s are excluded from analysis, estimates are the results of an ols regression with clustered robust standard errors (observations nested within individuals). | | | | | | |
| Table C2. *Explaining support for the use of referendums between and within respondents using multilevel regression* | | | | | | |
|  | *coef* | | *p* | | *robust SE* | |
| *within level:* |  | |  | |  | |
| majority perception **within** (ref: no) | **.51** | | **.000** | | **.04** | |
| sup. for policy proposal **within** | **.09** | | **.000** | | **.01** | |
|  |  | |  | |  | |
| *between level:* | **1.04** | | **.000** | | **.16** | |
| majority perception **between** (ref: no) | **.43** | | **.000** | | **.05** | |
| sup. for policy proposal **between** | **-.33** | | **.000** | | **.03** | |
| Political trust | **.30** | | **.000** | | **.04** | |
| Stealth Democracy | **-.06** | | **.014** | | **.02** | |
| Political Interest | **-.06** | | **.013** | | **.03** | |
| General trust | **.06** | | **.017** | | **.03** | |
| Risk aversion (reversed) | **.09** | | **.020** | | **.04** | |
| Universal values | .00 | | .130 | | .00 | |
| Age | .11 | | .126 | | .07 | |
| Gender (ref. male) |  | |  | |  | |
| Education (ref. low) |  | |  | |  | |
| middle | -.03 | | .777 | | .10 | |
| high | **-.31** | | **.002** | | **.10** | |
|  |  | |  | |  | |
|  |  | |  | |  | |
| Wald Chi2 (14) | 1055.60 | | .000 | |  | |
| N | 8847 | |  | |  | |
| *Note:* non-standardized coefficients are presented, estimates with a p-value below .05 are printed in bold. "don't know"s are exlcuded from analysis, estimates are the results of a multilevel regression with proposals nested within individuals | | | | | | |

| Table C3. *Characteristics of types of referendum supporters (mean scores)* | | | |
| --- | --- | --- | --- |
|  | *Full opponents* | *Switchers* | *Full supporters* |
| Political trust | 4.72 | 3.88 | 3.47 |
| General trust | 4.67 | 4.11 | 3.75 |
| Education (higher) | .68 | .51 | .38 |
| Emancipative values | 5.07 | 5.20 | 5.28 |
| Age | 48.83 | 50.51 | 52.96 |
| Political interest | 4.73 | 4.54 | 4.46 |
| gender (1=female) | .39 | .44 | .45 |
|  |  |  |  |
| N | 200 | 832 | 257 |
| *Note:* Full opponents include individuals that are neutral or against referendums across all policy proposals, Full supporters include individuals that are neutral or in favor of referendums on all policy proposals. Switchers include all others. N=1289 | | | |

| Table C4. *Explaining support for the use of referendums between and within respondents (bivariate analysis)* | | | | | | | | |
| --- | --- | --- | --- | --- | --- | --- | --- | --- |
|  | Model I | | Model II | | Model III | | Model IV | |
|  | *coef* | *p* | *coef* | *p* | *coef* | *p* | *coef* | *p* |
| *within level:* |  |  |  |  |  |  |  |  |
| majority perception **within** (ref: no) | **.56** | **.000** |  |  |  |  |  |  |
| sup. for policy proposal **within** |  |  | **1.47** | **.000** |  |  |  |  |
|  |  |  |  |  |  |  |  |  |
| *between level:* |  |  |  |  |  |  |  |  |
| majority perception **between** (ref: no) |  |  |  |  | **1.95** | **.000** |  |  |
| sup. for policy proposal **between** |  |  |  |  |  |  | **.61** | **.000** |
|  |  |  |  |  |  |  |  |  |
| Proposals (dummy, ref: home care) |  |  |  |  |  |  |  |  |
| cars in center | **-.43** | **.000** | **-.39** | **.000** | **-.41** | **.000** | **-.41** | **.000** |
| more police | **-.21** | **.002** | **-.24** | **.000** | **-.21** | **.004** | **-.22** | **.003** |
| social housing | -.01 | .920 | -.02 | .691 | .02 | .766 | .02 | .820 |
| taxes | **-.56** | **.000** | **-.49** | **.000** | **-.58** | **.000** | **-.59** | **.000** |
| own risk | **.15** | **.048** | **.28** | **.000** | **.18** | **.024** | **.18** | **.026** |
| migrants | .06 | .457 | .04 | .574 | -.05 | .552 | -.05 | .532 |
| abortion | **-.54** | **.000** | **-.54** | **.000** | **-.65** | **.000** | **-.66** | **.000** |
| welfare homeless | **-.74** | **.000** | **-.74** | **.000** | **-.84** | **.000** | **-.85** | **.000** |
| pension | **-.27** | **.000** | **-.17** | **.010** | **-.28** | **.000** | **-.28** | **.000** |
|  |  |  |  |  |  |  |  |  |
| R2 between | .01 |  | .01 |  | .10 |  | .12 |  |
| R2 within | .06 |  | .07 |  | .05 |  | .05 |  |
| R2 total | .04 |  | .04 |  | .07 |  | .09 |  |
| N | 8847 |  | 8847 |  | 8847 |  | 8847 |  |
| *Note:* non-standardized coefficients are presented, estimates with a p-value below .05 are printed in bold. "don't know"s are exlcuded from analysis, estimates are the results of an ols regression with clustered robust standard errors (observations nested within individuals). | | | | | | | | |

| Table C5. *Fixed effects model explaining support for referendums across proposals within respondents* | | | |
| --- | --- | --- | --- |
|  |  | | |
|  | *coef* | *p* | *robust SE* |
| majority perceptions (within) | **.50** | **.000** | **.05** |
| sup. for policy proposal (within) | **.13** | **.000** | **.01** |
|  |  |  |  |
| Proposals (dummy, ref: home care) |  |  |  |
| taxes | **-.67** | **.000** | **.07** |
| own risk | **-.42** | **.000** | **.07** |
| migrants | **-.28** | **.000** | **.08** |
| abortion | **-.79** | **.000** | **.08** |
| welfare homeless | -.11 | .159 | .08 |
| pension | **.17** | **.033** | **.08** |
| cars in center | **-.51** | **.000** | **.09** |
| more police | **-.82** | **.000** | **.08** |
| social housing | **-.52** | **.000** | **.08** |
|  |  |  |  |
| R2 | .10 |  |  |
| N | 8847 |  |  |
| *Note:* non-standardized coefficients are presented, estimates with a p-value below .05 are printed in bold. Don't knows are excluded from analysys. Estimates are the results of an ols regression with fixed effects and clustered robust standard errors (observations nested within individuals). | | | |

| Table C6. *Interaction between support for policy proposal and majority perceptions* | | | | | | |
| --- | --- | --- | --- | --- | --- | --- |
|  | Model I | | | Model II | | |
|  | *coef* | *robust SE* | *p* | *coef* | *robust SE* | *p* |
| *within level:* |  |  |  |  |  |  |
| majority perception **within** (ref: no) | .52 | .05 | .000 | .50 | .05 | .000 |
| sup. for policy proposal **within** | .13 | .01 | .000 | .13 | .01 | .000 |
|  |  |  |  |  |  |  |
| Interaction: mp within x sup. policy proposal within | .04 | .03 | .153 |  |  |  |
|  |  |  |  |  |  |  |
| *between level:* |  |  |  |  |  |  |
| majority perception **between** (ref: no) | 1.03 | .17 | .000 | .12 | .94 | .902 |
| sup. for policy proposal **between** | .43 | .05 | .000 | .08 | .38 | .827 |
|  |  |  |  |  |  |  |
| Interaction: mp between x sup. policy proposal between |  |  |  | .20 | .22 | .351 |
|  |  |  |  |  |  |  |
| Political trust | -.33 | .04 | .000 | -.33 | .04 | .000 |
| Stealth Democracy | 0.3 | .04 | .000 | .30 | .04 | .000 |
| Political Interest | -.06 | .03 | .029 | -.06 | .03 | .023 |
| General trust | -.10 | .03 | .000 | -.06 | .03 | .013 |
| Risk aversion (reversed) | .09 | .03 | .001 | .06 | .03 | .020 |
| Universal values | .10 | .04 | .022 | .09 | .04 | .035 |
| Age | .00 | .00 | .502 | .00 | .00 | .162 |
| Gender (ref. male) | .10 | .07 | .162 | .10 | .07 | .165 |
| Education (ref. low) |  |  |  |  |  |  |
| middle | -.06 | .10 | .548 | -.03 | .10 | .785 |
| high | -.43 | .10 | .000 | -.31 | .10 | .00 |
|  |  |  |  |  |  |  |
| Proposals (dummy, ref: cars in center |  |  |  |  |  |  |
| taxes | -.81 | .08 | .000 | -.80 | .08 | .000 |
| own risk | -.13 | .08 | .095 | -.12 | .08 | .145 |
| migrants | .16 | .08 | .047 | .16 | .08 | .042 |
| abortion | -.52 | .09 | .000 | -.51 | .09 | .000 |
| welfare homeless | -.83 | .08 | .000 | -.83 | .08 | .000 |
| pension | -.53 | .08 | .000 | -.52 | .08 | .000 |
| more police | -.69 | .07 | .000 | -.68 | .07 | .000 |
| social housing | -.43 | .07 | .000 | -.42 | .07 | .000 |
| home care | -.29 | .08 | .000 | -.29 | .08 | .000 |
|  |  |  |  |  |  |  |
| R between | .41 |  |  | .41 |  |  |
| R2 within | .08 |  |  | .08 |  |  |
| R2 total | .25 |  |  | .25 |  |  |
| N | 8847 |  |  | 8847 |  |  |
| *Note:* non-standardized coefficients are presented, estimates with a p-value below .05 are printed in bold. "don't know"s are excluded from analysis, estimates are the results of an ols regression with clustered robust standard errors issues nested within individuals). | | | | | | |

| Table C7. *Moderation of majority perceptions and societal issue salience* | | | | | | |
| --- | --- | --- | --- | --- | --- | --- |
|  | coef | robust SE | p | coef | robust SE | p |
|  | Model I | | | Model II | | |
| within level: |  |  |  |  |  |  |
| majority perception **within** (ref: no) | **.53** | **.06** | **.000** | **.21** | **.10** | **.038** |
| sup. for policy proposal **within** | **.14** | **.02** | **.000** | **.15** | **.02** | **.000** |
| issue salience (ref: low) |  |  |  |  |  |  |
| middle | -.61 | .31 | .050 | .12 | .07 | .084 |
| high | -.04 | .37 | .908 | **1.09** | **.08** | **.000** |
| Interaction: mp within x salience |  |  |  |  |  |  |
| middle |  |  |  | .20 | .12 | .106 |
| high |  |  |  | **.74** | **.15** | **.000** |
| between level: |  |  |  |  |  |  |
| majority perception **between** (ref: no) | **.73** | **.21** | **.001** | **1.10** | **.17** | **.000** |
| Interaction: mp between x salience |  |  |  |  |  |  |
| middle | **.42** | **.18** | **.018** |  |  |  |
| high | **.60** | **.21** | **.004** |  |  |  |
|  |  |  |  |  |  |  |
| sup. for policy proposal **between** | **.46** | **.05** | **.000** | **.47** | **.05** | **.000** |
| Political trust | **-.35** | **.04** | **.000** | **-.34** | **.04** | **.000** |
| Stealth Democracy | **.32** | **.04** | **.000** | **.32** | **.04** | **.000** |
| Political Interest | **-.06** | **.03** | **.016** | **-.06** | **.03** | **.016** |
| General trust | **-.06** | **.03** | **.022** | **-.06** | **.03** | **.024** |
| Risk aversion (reversed) | **.06** | **.03** | **.021** | **.06** | **.03** | **.023** |
| Universal values | .07 | .05 | .106 | .07 | .05 | .119 |
| Age | .00 | .00 | .235 | .00 | .00 | .216 |
| Gender (ref. male) | .07 | .08 | .362 | .07 | .08 | .371 |
| Education (ref. low) |  |  |  |  |  |  |
| middle | -.03 | .10 | .773 | -.03 | .10 | .763 |
| high | **-.32** | **.10** | **.00** | **-.31** | **.10** | **.00** |
|  |  |  |  |  |  |  |
| Proposals (dummy, ref: social housing) |  |  |  |  |  |  |
| more police | **.38** | **.07** | **.000** | **.39** | **.07** | **.000** |
| health care | **-.33** | **.08** | **.000** | **-.46** | **.08** | **.000** |
| welfare unemployed | **-.12** | **.07** | **.106** | -.14 | .07 | .054 |
| pensions | **.16** | **.07** | **.020** | **.15** | **.07** | **.028** |
|  |  |  |  |  |  |  |
| R^2^ between | .42 |  |  | .42 |  |  |
| R^2^ within | .09 |  |  | .10 |  |  |
| R^2^ total | .27 |  |  | .27 |  |  |
| N | 6487 |  |  | 6487 |  |  |
| Note: non-standardized coefficients are presented, estimates with a p-value below .05 are printed in bold. "don't know"s are excluded from analysis, estimates are the results of an ols regression with clustered robust standard errors (proposals nested within individuals). Some proposals were excluded because of lack of information on their salience level (abortion, cars in center, home care). Dummies for proposal related to taxes and migration were excluded due to multicollinearity. | | | | | | |

| Table C8: *The moderating effect of certainty on the relationship between majority perceptions and support for referendums (national)* | | | |
| --- | --- | --- | --- |
|  | coef | p | robust SE |
| within level: |  |  |  |
| majority perception **within** (ref: no) | **.56** | **.000** | **.06** |
| sup. for policy proposal **within** | **.12** | **.000** | **.02** |
|  |  |  |  |
| between level: |  |  |  |
| Certainty about mp | 1.08 | .060 | .57 |
| majority perception **between** (ref: no) | .14 | .562 | .24 |
|  |  |  |  |
| Interaction: mp between x certainty | -.01 | .939 | .14 |
|  |  |  |  |
| sup. for policy proposal **between** | **.41** | **.000** | **.05** |
| Political trust | **-.33** | **.000** | **.04** |
| Stealth Democracy | **.30** | **.000** | **.04** |
| Political Interest | **-.08** | **.004** | **.03** |
| General trust | **-.07** | **.018** | **.03** |
| Risk aversion (reversed) | .05 | .074 | .03 |
| Universal values | .07 | .112 | .04 |
| Age | .00 | .510 | .00 |
| Gender (ref. male) | .10 | .190 | .08 |
| Education (ref. low) |  |  |  |
| middle | .00 | .963 | .11 |
| high | -.27 | .011 | .11 |
|  |  |  |  |
| Proposals (dummy, ref: social housing) |  |  |  |
| taxes | **-.18** | **.861** | **1.05** |
| health care | **.50** | **.632** | **1.05** |
| migration | **.77** | **.459** | **1.05** |
| abortion | **.10** | **.921** | **1.05** |
| welfare unemployed | -.22 | .833 | 1.04 |
| pensions | .09 | .929 | 1.05 |
|  |  |  |  |
| R2 between | .40 |  |  |
| R2 within | .11 |  |  |
| R2 total | .27 |  |  |
| N | 5444 |  |  |
| Note: non-standardized coefficients are presented, estimates with a p-value below .05 are printed in bold. "don't know"s are excluded from analysis, estimates are the results of an OLS regression with clustered robust standard errors (observations nested within individuals). | | | |

| Table C9: *The moderating effect of certainty on the relationship between majority perceptions and support for referendums (local)* | | | |
| --- | --- | --- | --- |
|  | coef | p | robust SE |
| within level: |  |  |  |
| majority perception **within** (ref: no) | **.36** | **.000** | **.08** |
| sup. for policy proposal **within** | **.15** | **.000** | **.02** |
|  |  |  |  |
| between level: |  |  |  |
| Certainty about mp | .11 | .737 | .33 |
| majority perception **between** (ref: no) | .64 | .447 | .84 |
|  |  |  |  |
| Interaction: mp between x certainty | .02 | .914 | .19 |
|  |  |  |  |
| sup. for policy proposal **between** | **.44** | **.000** | **.07** |
| Political trust | **-.31** | **.000** | **.05** |
| Stealth Democracy | **.26** | **.000** | **.05** |
| Political Interest | **-.07** | **.062** | **.04** |
| General trust | -.03 | .387 | .03 |
| Risk aversion (reversed) | .05 | .123 | .03 |
| Universal values | .08 | .198 | .06 |
| Age | -.01 | .021 | .00 |
| Gender (ref. male) | .15 | .106 | .10 |
| Education (ref. low) |  |  |  |
| middle | -.01 | .915 | .13 |
| high | **-.29** | **.025** | **.13** |
|  |  |  |  |
| Proposals (dummy, ref: cars in center) |  |  |  |
| more police | **-.66** | **.000** | **.07** |
| social housing | **-.40** | **.000** | **.07** |
| home care | **-.28** | **.001** | **.08** |
|  |  |  |  |
| R2 between | .27 |  |  |
| R2 within | .06 |  |  |
| R2 total | .22 |  |  |
| N | 3403 |  |  |
| Note: non-standardized coefficients are presented, estimates with a p-value below .05 are printed in bold. "don't know"s are excluded from analysis, estimates are the results of an OLS regression with clustered robust standard errors (observations nested within individuals). | | | |

| Table C10. Moderation of majority perceptions and support for policy change with education levels | | | | | | |
| --- | --- | --- | --- | --- | --- | --- |
|  | Model I | | | Model II | | |
|  | *coef* | *robust SE* | *p* | *coef* | *robust SE* | *p* |
| *within level:* |  |  |  |  |  |  |
| majority perception **within** (ref: no) | **.73** | **.12** | **.000** | **.50** | **.05** | **.000** |
| sup. for policy proposal **within** | **.13** | **.01** | **.000** | **.20** | **.03** | **.000** |
|  |  |  |  |  |  |  |
| Interaction: mp **within** x education |  |  |  |  |  |  |
| middle | -.19 | .15 | .218 |  |  |  |
| high | **-.32** | **.13** | **.014** |  |  |  |
|  |  |  |  |  |  |  |
| Interaction: pref. for policy **within** x education |  |  |  |  |  |  |
| middle |  |  |  | **-.10** | **.03** | **.002** |
| high |  |  |  | **-.07** | **.03** | **.023** |
|  |  |  |  |  |  |  |
| *between level:* |  |  |  |  |  |  |
| majority perception **between** (ref: no) | **1.02** | **.17** | **.000** | **1.02** | **.17** | **.000** |
| sup. for policy proposal **between** | **.43** | **.05** | **.000** | **.43** | **.05** | **.000** |
| Political trust | **-.33** | **.04** | **.000** | **-.33** | **.04** | **.000** |
| Stealth Democracy | **.30** | **.04** | **.000** | **.30** | **.04** | **.000** |
| Political Interest | **-.06** | **.03** | **.023** | **-.06** | **.03** | **.023** |
| General trust | **-.06** | **.03** | **.014** | **-.06** | **.03** | **.014** |
| Risk aversion (reversed) | **.06** | **.03** | **.018** | **.06** | **.03** | **.018** |
| Universal values | .09 | .04 | .035 | .09 | .04 | .037 |
| Age | .00 | .00 | .157 | .00 | .00 | .150 |
| Gender (ref. male) | .10 | .07 | .170 | .10 | .07 | .170 |
| Education (ref. low) |  |  |  |  |  |  |
| middle | -.03 | .10 | .758 | -.02 | .10 | .859 |
| high | **-.31** | **.10** | **.00** | **-.30** | **.10** | **.00** |
|  |  |  |  |  |  |  |
| Proposals (dummy, ref: cars in center |  |  |  |  |  |  |
| taxes | -.80 | .08 | .000 | -.80 | .08 | .000 |
| own risk | -.13 | .08 | .114 | -.12 | .08 | .128 |
| migrants | .16 | .08 | .051 | .17 | .08 | .038 |
| abortion | -.51 | .09 | .000 | -.51 | .09 | .000 |
| welfare homeless | -.83 | .08 | .000 | -.83 | .08 | .000 |
| pension | -.53 | .08 | .000 | -.53 | .08 | .000 |
| more police | -.68 | .07 | .000 | -.68 | .07 | .000 |
| social housing | -.43 | .07 | .000 | -.42 | .07 | .000 |
| home care | -.29 | .08 | .000 | -.29 | .08 | .000 |
|  |  |  |  |  |  |  |
| R between | .40 |  |  | .40 |  |  |
| R2 within | .09 |  |  | .09 |  |  |
| R2 total | .25 |  |  | .25 |  |  |
| N | 8847 |  |  | 8847 |  |  |
| *Note:* non-standardized coefficients are presented, estimates with a p-value below .05 are printed in bold. "don't know"s are excluded from analysis, estimates are the results of an ols regression with clustered robust standard errors (observations nested within individuals). | | | | | | |

| Table C11: *Regression model for general preferences for a referendum on the national and local level* | | | | | | |
| --- | --- | --- | --- | --- | --- | --- |
|  | **national** | | | **local** | | |
|  | *coef.* | *p* | *SE* | *coef.* | *p* | *SE* |
| Majority perceptions | **.48** | **.00** | **.035** | **.48** | **.00** | **.012** |
| Political trust | **-.33** | **.00** | **.041** | **-.33** | **.00** | **.015** |
| Stealth Democracy | **.38** | **.00** | **.046** | **.34** | **.00** | **.016** |
| Political Interest | .00 | .98 | .032 | .02 | .05 | .012 |
| General trust | **-.04** | **.20** | **.033** | **-.04** | **.00** | **.012** |
| Risk aversion | .03 | .31 | .033 | .04 | .00 | .012 |
| Universal Values | **.09** | **.07** | **.050** | **.14** | **.00** | **.018** |
| Age | -.01 | .06 | .003 | .00 | .43 | .001 |
| Gender (1=female) | -.04 | .67 | .092 | .12 | .00 | .033 |
| Education (low) |  |  |  |  |  |  |
| middle | -.06 | .63 | .131 | .12 | .01 | .046 |
| high | **-.22** | **.09** | **.130** | .00 | .98 | .046 |
| R2 | .34 |  |  | .32 |  |  |
| N | 1064 |  |  | 1039 |  |  |
| *Note:* Estimates with a p-value below .05 are printed in bold. "don't know"s are exlcuded from analysis, estimates are the result of a OLS regression. | | | | | | |
|  | | | | | | |

| Table C12: *Explaining support for the use of referendums between and within respondents controlling for general preferences for referendums* | | | |
| --- | --- | --- | --- |
|  | *coef* | *p* | *robust SE* |
| *within level:* |  |  |  |
| majority perception **within** (ref: no) | **.50** | **.000** | **.05** |
| sup. for policy proposal **within** | **.13** | **.000** | **.01** |
| *between level:* |  |  |  |
| majority perception **between** (ref: no) | **.79** | **.000** | **.15** |
| sup. for policy proposal **between** | **.34** | **.000** | **.04** |
| General pref. for referendums | **.31** | **.000** | **.02** |
| Political trust | **-.23** | **.000** | **.03** |
| Stealth Democracy | **.16** | **.000** | **.04** |
| Political Interest | **-.06** | **.010** | **.02** |
| General trust | **-.06** | **.020** | **.03** |
| Risk aversion (reversed) | .04 | .068 | .02 |
| Universal values | .07 | .107 | .05 |
| Age | .00 | .458 | .00 |
| Gender (ref. male) | .09 | .206 | .07 |
| Education (ref. low) |  |  |  |
| middle | -.03 | .722 | .09 |
| high | **-.25** | **.007** | **.09** |
|  |  |  |  |
| Proposals (dummy, ref: home care) |  |  |  |
| cars in center | **-.67** | **.000** | **.07** |
| more police | **-.42** | **.000** | **.07** |
| social housing | **-.29** | **.000** | **.08** |
| taxes | **-.80** | **.000** | **.08** |
| own risk | -.11 | .151 | .08 |
| migrants | **.16** | **.042** | **.08** |
| abortion | **-.51** | **.000** | **.09** |
| welfare homeless | **-.82** | **.000** | **.08** |
| pension | **-.52** | **.000** | **.08** |
|  |  |  |  |
| R between | .50 |  |  |
| R2 within | .08 |  |  |
| R2 total | .30 |  |  |
| N | 8847 |  |  |
| *Note:* non-standardized coefficients are presented, estimates with a p-value below .05 are printed in bold. "don't know"s are exlcuded from analysis, estimates are the results of an ols regression with clustered robust standard errors (observations nested within individuals). | | | |

| Table C13. *Explaining support for the use of referendums including “don’t know” responses for majority perceptions* | | | |
| --- | --- | --- | --- |
|  | *coef* | *p* | *robust SE* |
| majority perception (ref. no) |  |  |  |
| yes | **.65** | **.000** | **.06** |
| don't know | .03 | .685 | .07 |
| sup. for policy proposal | **.19** | **.000** | **.01** |
| Political trust | **-.32** | **.000** | **.03** |
| Stealth Democracy | **.32** | **.000** | **.04** |
| Political Interest | **-.06** | **.025** | **.02** |
| General trust | **-.06** | **.013** | **.02** |
| Risk aversion (reversed) | **.06** | **.016** | **.02** |
| Universal values | **.12** | **.003** | **.04** |
| Age | .00 | .894 | .00 |
| Gender (ref. male) | **.14** | **.047** | **.07** |
| Education (ref. low) |  |  |  |
| middle | -.07 | .490 | .10 |
| high | **-.35** | **.000** | **.09** |
|  |  |  |  |
| Proposals (dummy, ref: home care) |  |  |  |
| cars in center | **-.76** | **.000** | **.06** |
| more police | **-.51** | **.000** | **.06** |
| social housing | **-.43** | **.000** | **.06** |
| taxes | **-.87** | **.000** | **.07** |
| own risk | **-.23** | **.001** | **.07** |
| migrants | **.23** | **.001** | **.07** |
| abortion | **-.38** | **.000** | **.07** |
| welfare unemployed | **-.76** | **.000** | **.07** |
| pension | **-.57** | **.000** | **.07** |
|  |  |  |  |
| R2 | .23 |  |  |
| N | 11560 |  |  |
| *Note:* non-standardized coefficients are presented, estimates with a p-value below .05 are printed in bold. "don't know"s are excluded from analysis, estimates are the results of an ols regression with clustered robust standard errors (proposals nested within individuals). | | | |

| Table C14. *Explaining proposal specific preferences for referendums (national level)* | | | | | | | | | | | | | | | | | | |
| --- | --- | --- | --- | --- | --- | --- | --- | --- | --- | --- | --- | --- | --- | --- | --- | --- | --- | --- |
| Referendum on… | Taxes | | | Health Care | | | Immigration | | | Abortion | | | Unemployment benefits | | | Pensions | | |
|  | coef | r. SE | p | coef | r. SE | p | coef | r. SE | p | coef | r. SE | p | coef | r. SE | p | coef | r. SE | p |
| majority perception (ref. no) |  |  |  |  |  |  |  |  |  |  |  |  |  |  |  |  |  |  |
| yes | .26 | .13 | .051 | .82 | .17 | .000 | .41 | .13 | .001 | .31 | .14 | .031 | .64 | .12 | .000 | .42 | .15 | .005 |
| don't know | -.11 | .16 | .504 | .21 | .20 | .295 | .07 | .15 | .648 | .08 | .13 | .569 | .24 | .13 | .053 | .10 | .17 | .570 |
| Sup. for policy proposal | .23 | .03 | .000 | .31 | .03 | .000 | -.19 | .04 | .000 | .18 | .03 | .000 | .29 | .03 | .000 | .31 | .03 | .000 |
|  |  |  |  |  |  |  |  |  |  |  |  |  |  |  |  |  |  |  |
| Political trust | -.30 | .05 | .000 | -.32 | .05 | .000 | -.41 | .05 | .000 | -.18 | .05 | .000 | -.28 | .04 | .000 | -.36 | .04 | .000 |
| Stealth Democracy | .31 | .05 | .000 | .34 | .05 | .000 | .36 | .05 | .000 | .25 | .06 | .000 | .28 | .05 | .000 | .36 | .05 | .000 |
| Political Interest | -.13 | .04 | .000 | -.07 | .04 | .063 | .05 | .04 | .212 | -.05 | .04 | .184 | -.07 | .03 | .042 | -.11 | .03 | .001 |
| General trust | -.05 | .04 | .185 | .00 | .04 | .989 | -.09 | .04 | .014 | -.09 | .04 | .032 | -.09 | .04 | .014 | -.07 | .04 | .059 |
| Risk aversion (reversed) | .13 | .04 | .001 | .09 | .04 | .014 | .03 | .04 | .489 | .02 | .04 | .564 | .07 | .04 | .049 | .07 | .04 | .042 |
| Universal values | .14 | .06 | .013 | .18 | .06 | .001 | .00 | .06 | .972 | .10 | .06 | .090 | .02 | .05 | .652 | .10 | .05 | .058 |
| Age | .00 | .00 | .958 | .00 | .00 | .476 | .00 | .00 | .951 | .00 | .00 | .887 | .00 | .00 | .527 | .00 | .00 | .260 |
| Gender (ref. male) | .26 | .10 | .011 | .19 | .10 | .074 | .05 | .11 | .649 | .18 | .11 | .104 | -.09 | .10 | .342 | -.03 | .10 | .758 |
| Education (ref. low) |  |  |  |  |  |  |  |  |  |  |  |  |  |  |  |  |  |  |
| middle | -.13 | .15 | .372 | -.24 | .15 | .111 | -.01 | .15 | .951 | -.09 | .16 | .568 | .20 | .14 | .155 | -.10 | .14 | .466 |
| high | -.56 | .14 | .000 | -.32 | .15 | .032 | -.15 | .15 | .313 | -.23 | .16 | .151 | -.16 | .14 | .264 | -.42 | .14 | .003 |
| R2 | .24 |  |  | .34 |  |  | .26 |  |  | .10 |  |  | .24 |  |  | .33 |  |  |
| N | 1203 |  |  | 1203 |  |  | 1203 |  |  | 1203 |  |  | 1203 |  |  | 1203 |  |  |
| Note: unstandardized coefficients are presented, estimates are the results of a OLS regression | | | | | | | | | | | | | | | | | | |

| Table C15. *Explaining proposal specific preferences for referendums (local level)* | | | | | | | | | | | | |
| --- | --- | --- | --- | --- | --- | --- | --- | --- | --- | --- | --- | --- |
| Referendum on… | Cars in city center | | | More police | | | Social housing | | | Home care | | |
|  | coef | r. SE | p | coef | r. SE | p | coef | r. SE | p | coef | r. SE | p |
| majority perception (ref. no) |  |  |  |  |  |  |  |  |  |  |  |  |
| yes | .30 | .15 | .040 | .56 | .19 | .004 | .12 | .15 | .423 | .52 | .17 | .003 |
| don't know | -.35 | .16 | .034 | -.02 | .20 | .911 | -.37 | .16 | .020 | -.10 | .18 | .565 |
| Sup. for policy proposal | -.02 | .03 | .371 | .26 | .04 | .000 | .39 | .03 | .000 | .34 | .04 | .000 |
|  |  |  |  |  |  |  |  |  |  |  |  |  |
| Political trust | -.25 | .05 | .000 | -.31 | .05 | .000 | -.38 | .05 | .000 | -.37 | .05 | .000 |
| Stealth Democracy | .38 | .06 | .000 | .24 | .05 | .000 | .27 | .05 | .000 | .29 | .05 | .000 |
| Political Interest | -.01 | .04 | .751 | -.08 | .04 | .042 | -.09 | .04 | .009 | -.07 | .03 | .032 |
| General trust | -.06 | .04 | .157 | -.12 | .04 | .002 | -.03 | .04 | .376 | -.01 | .03 | .855 |
| Risk aversion (reversed) | .09 | .04 | .031 | .07 | .04 | .074 | .02 | .04 | .581 | .02 | .03 | .531 |
| Universal values | .16 | .06 | .011 | .13 | .06 | .034 | .08 | .06 | .181 | .15 | .05 | .005 |
| Age | .00 | .00 | .445 | .00 | .00 | .926 | .00 | .00 | .464 | .00 | .00 | .801 |
| Gender (ref. male) | .30 | .11 | .010 | .15 | .11 | .171 | .13 | .10 | .224 | .19 | .10 | .060 |
| Education (ref. low) |  |  |  |  |  |  |  |  |  |  |  |  |
| middle | .06 | .16 | .727 | -.10 | .16 | .530 | -.02 | .15 | .866 | -.19 | .14 | .179 |
| high | -.09 | .16 | .585 | -.40 | .15 | .011 | -.24 | .15 | .098 | -.42 | .14 | .003 |
| R2 | .13 |  |  | .23 |  |  | .27 |  |  | .32 |  |  |
| N | 1203 |  |  | 1203 |  |  | 1203 |  |  | 1203 |  |  |
| Note: unstandardized coefficients are presented, estimates are the results of a OLS regression | | | | | | | | | | | | |

Table C16: S*upport for policy proposal and majority perception for “More migrants should be admitted to the Netherlands”*

|  | Majority perceptions | |  |  |
| --- | --- | --- | --- | --- |
| Support for policy proposal | No | Yes | don't know | Total |
| No support - 1 | 81 | 236 | 44 | 361 |
| 2 | 30 | 118 | 30 | 178 |
| 3 | 46 | 83 | 54 | 183 |
| 4 | 111 | 64 | 69 | 244 |
| 5 | 90 | 18 | 18 | 126 |
| 6 | 51 | 8 | 12 | 71 |
| complete support - 7 | 29 | 5 | 6 | 40 |
| Total | 438 | 532 | 233 | 1,203 |
